# Autoantibody Reactome Profiling Reveals Distinct Humoral Immune Signatures Induced by Simulated Microgravity, Radiation, and Combined Exposure in Mice

**DOI:** 10.1016/j.mcpro.2026.101665

**Published:** 2026-09-18

**Authors:** Lining Wu, Kaipeng Zheng, Bomiao Yu, Bingxin Gao, Pancheng Xiao, Saiya Wang, Yanjun Li, Ruimin Liu, Xiaomei Zhang, Mansheng Li, Chun-Ping Cui, Weiming Tian, Xiaobo Yu

**Affiliations:** 1 School of Basic Medical Sciences, Anhui Medical University, Hefei, Anhui, China; 2 State Key Laboratory of Medical Proteomics, Beijing Proteome Research Center, National Center for Protein Sciences (Beijing), Beijing Institute of Lifeomics, Beijing, China; 3 School of Life Science and Technology, Harbin Institute of Technology, Harbin, Heilongjiang, China; 4 ProteomicsEra Medical Co, Ltd, Beijing, China

**Keywords:** autoantibody reactome, autoantigen microarray, immune dysfunction, microgravity, space radiation

## Abstract

Microgravity (μG) and space radiation are major environmental stressors leading to immune dysfunction in astronauts, but the mechanisms governing humoral immunity and effective interventions remain unclear. In this study, we used a high-throughput autoantigen microarray derived from the AAgAtlas 1.0 database to profile humoral immune responses in mice exposed to μG and proton irradiation. We generated 70,490 autoantibody reactivity measurements and observed combined effects of radiation and μG on humoral immunity. Specifically, 38 IgM and 57 IgG autoantibody candidates showed dose-associated changes across 0, 1 and 5 Gy proton irradiation. We further established a global autoantibody landscape for mice under μG, 1 Gy, 5 Gy and μG & 1 Gy. Autoantibody targets altered under μG were enriched for cardiovascular-associated proteins, whereas proton irradiation-associated targets were enriched for DNA repair-related proteins and co-exposure-associated targets were enriched for inflammation-related proteins, revealing distinct humoral immune recognition signatures across exposure conditions. Additionally, a glutamine-free diet (GFD) was associated with changes in autoantibody profiles and shifts in selected bone and hematological parameters toward control levels under μG and μG & 1 Gy conditions. This work provides a resource for investigating humoral immunity under space-related stress and identifies candidate autoantibody signatures and a potential role of dietary modulation that warrant further validation.

## Highlights

• A comprehensive IgM and IgG autoantibody reactome landscape was established under simulated microgravity and proton irradiation.
• Microgravity, proton irradiation and combined exposure induced distinct humoral immune recognition patterns.
• Candidate autoantibody signatures and dietary intervention-associated changes provide a framework for future space immunology studies.

## Graphical Abstract

**Figure:**
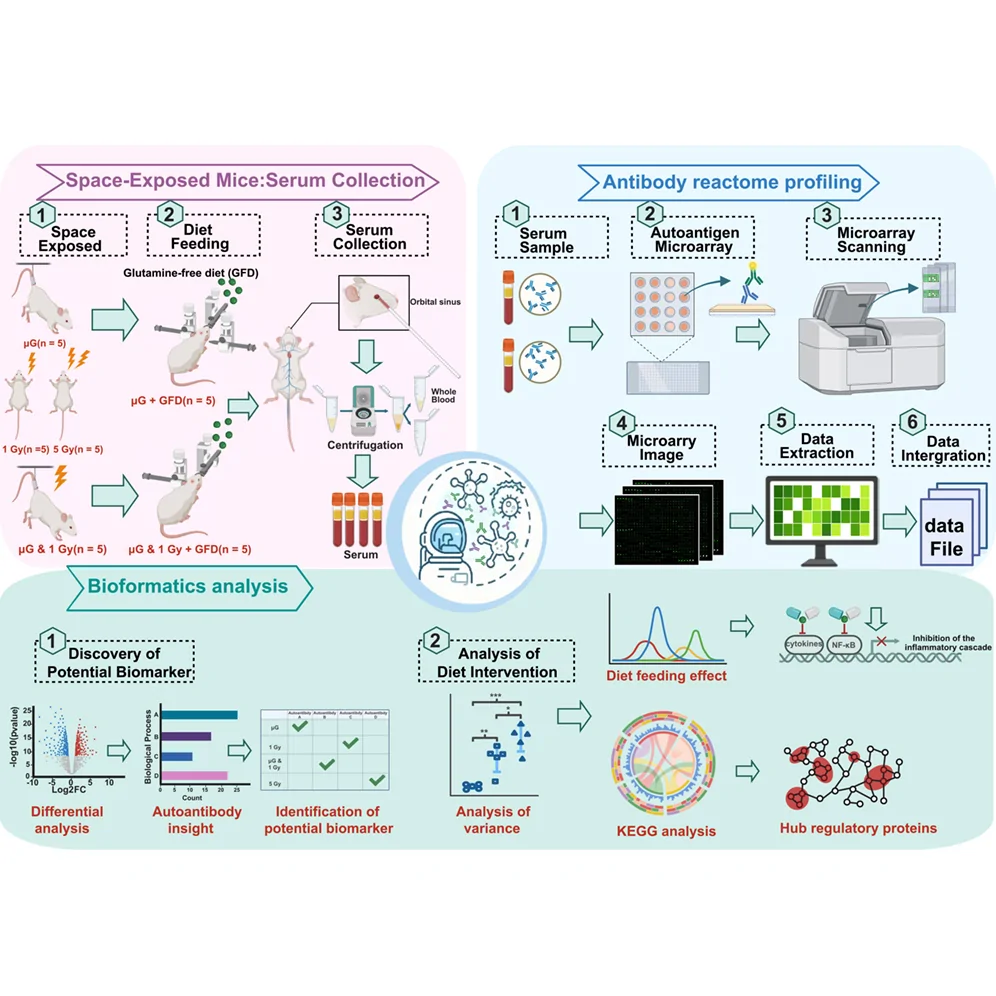

## In Brief

Space-related environmental stressors are associated with altered humoral immune recognition patterns. In this study, we used a high-throughput autoantigen microarray to construct an autoantibody reactivity landscape under simulated microgravity, proton irradiation, and combined exposure conditions. We identified distinct IgM/IgG autoantibody signatures associated with different space-related stressors and explored the potential association of glutamine-free diet intervention with altered autoantibody profiles.

As human space exploration continues to expand, crewed missions are becoming increasingly frequent (1, 2). During more than 50 years of spaceflight, numerous studies have demonstrated that the space environment can adversely affect biological systems (3, 4). Astronauts face multiple health risks, including mitochondrial dysfunction, immune dysfunction, vascular alterations, fluid shifts, cognitive impairment and genomic instability (5, 6). The space environment contains multiple stressors, including microgravity (μG), radiation, elevated CO_2_ levels, psychological stress, sleep deprivation, nutritional issues and others that could contribute to alterations in cognition and neural activity (7, 8). Among these, μG and radiation are two of the primary factors influencing astronaut health during space missions (9, 10). For example, medical data collected from 46 astronauts who spent 6 months aboard the International Space Station (ISS) revealed that 46% experienced immunological issues, such as skin sensitivity (1.12 cases per flight-year) and upper respiratory symptoms (0.97 cases per flight-year), accompanied by cold sores, ear problems and other infectious symptoms (over 0.3 cases per flight-year) (11, 12). This highlights the significant changes in human immunity induced by extreme space environments.

The perturbations induced by these extreme environments may disrupt immune homeostasis, wherein B cells may produce autoantibodies or activate other immune cells to erroneously target the body, resulting in the immune system mistakenly attacking normal tissue (13, 14). Furthermore, altered circulating autoantibody profiles have been reported in immunodeficiency and malignancy, thereby reflecting changes in humoral immunity (15, 16). Thus, investigation of the autoantibody reactome is of great significance for elucidating the mechanisms underlying humoral immune dysfunction caused by the extreme space environment. However, significant knowledge gaps remain: (*i*) the molecular mechanisms underlying the separate and combined effects of μG and space radiation on humoral immunity have yet to be systematically characterized; (*ii*) effective countermeasures against spaceflight-associated humoral immune dysfunction are still limited.

Antigen microarray technology provides a high-throughput proteomic approach for profiling autoantibodies in body fluids (17, 18). Previously, we developed a high-throughput autoantigen microarray based on the AAgAtlas 1.0 database, achieving high sensitivity and specificity, which enables large-scale analysis of humoral immune changes in cancer, autoimmune and infectious diseases using only 1 to 2 μl of serum or plasma (19, 20). Humoral immune responses involve both IgM and class-switched IgG antibodies, which have distinct and complementary roles in B-cell-mediated immunity (21, 22).

In this study, we performed comprehensive profiling of IgM and IgG autoantibody repertoires in 35 mice exposed to μG, proton irradiation (1/5 Gy) and a combined exposure (μG & 1 Gy). Moreover, our previous study showed that a glutamine-free diet (GFD) effectively attenuates bone loss under μG conditions (23). Given that μG may also impair humoral immunity, we investigated whether GFD was associated with shifts in μG-induced autoantibody profiles and further assessed its potential effects on humoral immune dysfunction. Our objective is to characterize the impact of μG and proton irradiation on humoral immunity, identify associated molecular signatures and explore GFD-associated modulation of these immune alterations as a potential dietary research direction.

## Experimental Procedures

### Mouse Models

Eight-week-old male SPF C57BL/6J mice (20 ± 2 g) were purchased from Liaoning Changsheng Biotechnology Co, Ltd. Mice were housed under controlled SPF conditions (22 ± 2 °C, 55% ± 10% humidity, and 12 h light/dark cycle) with free access to food and water. Control and experimental groups were maintained under identical housing conditions except for the specific treatments. After 7 days of acclimatization, mice were randomly assigned to seven groups (n = 5 per group): Control, μG, 1 Gy, 5 Gy, μG & 1 Gy, μG + GFD, and μG & 1 Gy + GFD.

Simulated microgravity was induced by tail suspension for 28 days with hindlimb unloading at a 30° head-down tilt angle, as previously described (24, 25). Whole-body proton irradiation was performed using a 150 MeV proton beam generated by a cyclotron at the Ground-based Space Environment Simulation Facility of Harbin Institute of Technology. Mice received a single exposure to 1 Gy or 5 Gy proton irradiation at a dose rate of 0.2 Gy/min. After irradiation, mice in the 1 Gy and 5 Gy groups were maintained under standard housing conditions for 28 days before sample collection. For combined exposure, irradiation was performed first, followed by a 24 h recovery period and subsequent 28-day simulated microgravity treatment. At the end of the respective treatment periods, serum and femur samples were collected; for the hindlimb-unloading groups, samples were collected immediately after termination of unloading. Detailed procedures are provided in the Supplementary Methods.

### Sample Collection

At the end of the respective treatment periods, blood samples were collected from all groups. For mice in the hindlimb unloading groups, blood collection was performed immediately after release from suspension. Blood was collected via orbital sinus puncture into anticoagulant-free tubes and allowed to clot completely at room temperature for 2 h. The samples were then centrifuged at 3000 rpm for 15 min at 4 °C. The serum was carefully collected, snap-frozen in liquid nitrogen, and stored at −80 °C until further analysis. All experimental procedures were approved by the Biosafety and Ethics Committee of Harbin Institute of Technology (Ethics Approval No. IACUC-2026015). Detailed information on the experimental grouping is provided in Supplemental Table S1.

### Autoantibody Profiling

The autoantigen microarray was manufactured by ProteomicsEra Medical Co, Ltd. (https://en.proteomics-era.com/) according to the AAgAtlas 1.0 database (26). The tissue localization of these autoantigens is shown in Supplemental Fig. S1*A*. The reproducibility of the microarray, both intra-assay and inter-assay, ranged from 0.90 to 0.96 (Supplemental Fig. S1*B*). The autoantigen microarray was provided by ProteomicsEra Medical Co, Ltd. For assay-level microarray-batch replication, a separately fabricated customized autoantigen microarray containing 217 autoantigens selected from candidate autoantibody signatures identified in the discovery-phase analysis was used to re-profile the same serum samples. The selected antigens included candidates with nominal *p* < 0.05 associated with μG and μG & 1 Gy exposure compared with the Control group, IgM and IgG candidates showing the strongest correlations with radiation dose across 0, 1 and 5 Gy irradiation and GFD-responsive candidates showing the strongest recovery trends toward the Control profile after dietary intervention.

Serum detection was performed as previously described (27). Donkey anti-mouse IgM and goat anti-mouse IgG were employed for secondary autoantibody detection at 4 μg/ml each. Fluorescent images were acquired using a GenePix 4300A scanner and signal intensities were extracted with GenePix Pro v7 software (Molecular Devices). Signal-to-Noise Ratio (SNR) was calculated following an established method (28).

### Bioinformatics Analysis

Bioinformatics analysis and data visualization were performed using R (v4.4.1; https://www.Rproject.org). Specifically, the Mfuzz package (v2.66.0) was used for clustering analysis, the org.Mm.eg.db package (v3.20.0) was used for gene annotation of mouse autoantigens corresponding to differential autoantibodies, and the clusterProfiler package (v4.14.6) was used for GO and KEGG enrichment analyses. The 1007 autoantigens represented on the microarray were used as the reference background for all GO and KEGG enrichment analyses. Within each enrichment analysis, *p* values across all tested GO terms or KEGG pathways were adjusted using the Benjamini-Hochberg procedure. Terms with BH-adjusted *p* < 0.05 were considered FDR-supported enrichment findings, whereas terms with nominal *p* < 0.05 but BH-adjusted *p* ≥ 0.05 were considered exploratory enrichment findings. Tissue localization data for the corresponding autoantigens were retrieved from the Human Protein Atlas (HPA) database (https://www.proteinatlas.org/). The workflow diagram was created in Adobe Illustrator using graphical elements sourced from BioRender. Created in BioRender. Lining, W. (2026) https://BioRender.com/1jyscov.

### Statistical Analysis

The SNR data for IgM and IgG autoantibodies targeting 1007 autoantigens were median-normalized and log_2_-transformed (Supplemental Fig. S1*C*). PCA using all detected autoantibodies was performed on the normalized and log_2_-transformed data, with features centered and scaled to unit variance, to assess the overall sample distribution (Supplemental Fig. S1, *D* and *E*). PCA based on nominally significant candidate autoantibodies was retained as a visualization of group-associated separation (Supplemental Fig. S2, *A* and *B*). Differential analysis of autoantibody reactivity profiles was performed using limma. For each prespecified biological comparison and antibody isotype, the antigen-level tests for all 1007 autoantigens were treated as one hypothesis-testing family and adjusted together using the Benjamini-Hochberg procedure. Autoantibodies with BH-adjusted *p* < 0.05 were classified as FDR-supported findings, whereas those with nominal *p* < 0.05 but BH-adjusted *p* ≥ 0.05 were retained only as exploratory candidates. For differential autoantibody analyses, effect size was reported as the estimated log_2_ fold change (log_2_FC) between the two groups.

## Results

### Simulated Microgravity and Proton Irradiation Are Associated with Bone Loss and Hematological Alterations

To assess the impact of spaceflight-related factors on immune function, we established mouse models subjected to simulated μG, 1 Gy, 5 Gy and μG & 1 Gy. For dietary intervention, additional groups were subjected to μG or μG & 1 Gy while receiving a GFD, generating the μG + GFD and μG & 1 Gy + GFD groups (Fig. 1*A*).

**Fig. 1:**
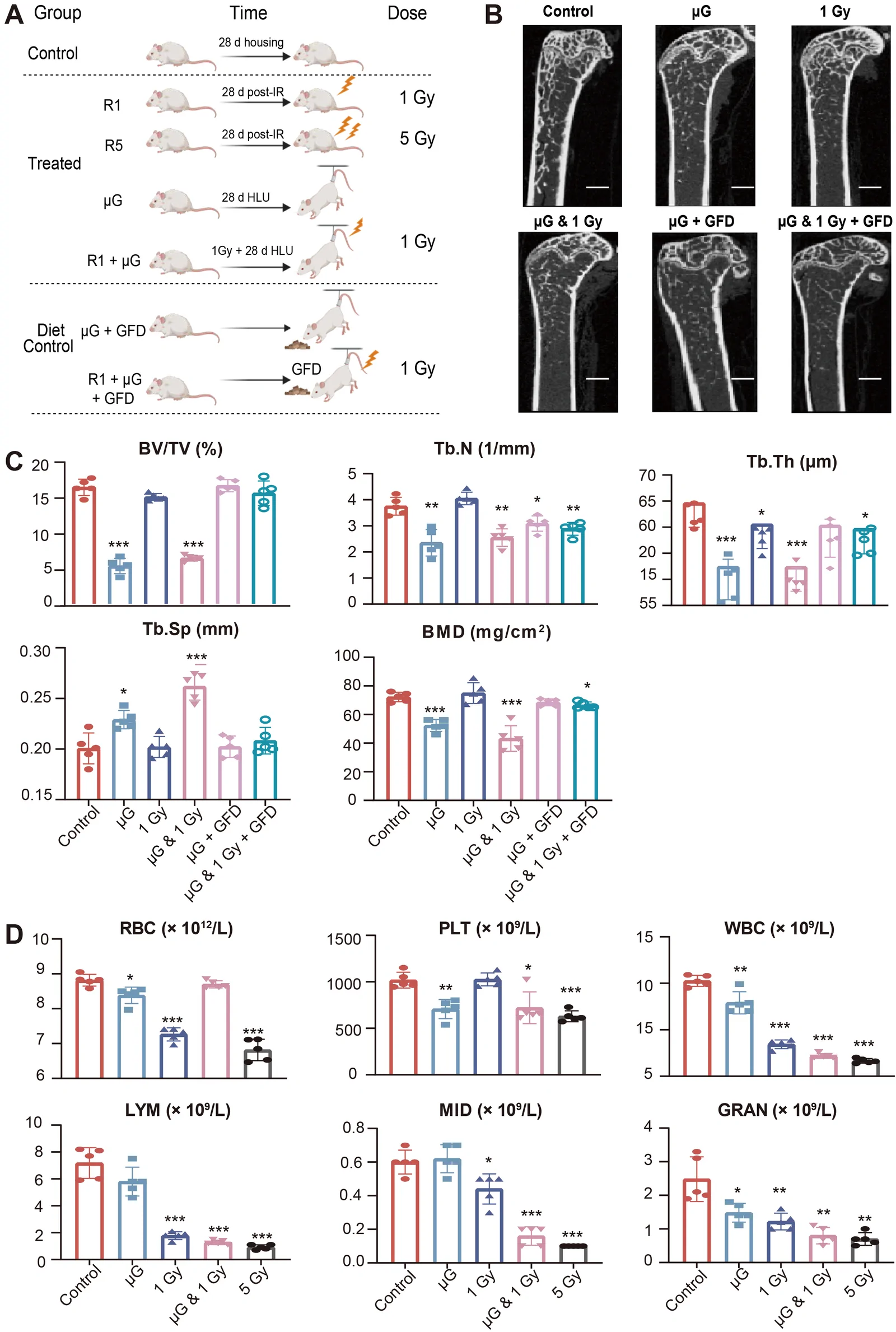
**Effects of different interventions and dietary factors on femoral bone microstructure and peripheral blood parameters in mice.***A*, schematic of the experimental design and timeline. R1 and R5 indicate single 1 Gy and 5 Gy whole-body proton irradiation, respectively, followed by 28 days of standard housing. μG indicates 28 days of hindlimb unloading. For combined exposure, 1 Gy irradiation was followed by 24 h recovery and 28 days of μG. GFD indicates glutamine-free diet. Samples were collected at the experimental endpoint. *B*, representative micro-CT images of the whole femoral bones. n = 5 per group, with a total of 35 mice across seven experimental groups. Scale bar, 0.5 mm. *C*, quantitative analysis of bone microstructural parameters, including BMD, BV/TV, Tb.N, Tb.Sp and Tb.Th. *D*, hematological parameters, including red blood cell count (RBC), platelet count (PLT), WBC, lymphocyte count (LYM), MID and GRAN. Data are presented as mean ± SEM. Statistical significance was assessed using unpaired two-tailed Student’s t-tests comparing each treatment group with the control group, with *p* values adjusted for multiple comparisons using the Benjamini-Hochberg method. ∗BH-adjusted *p* < 0.05, ∗∗BH-adjusted *p* < 0.01, ∗∗∗BH-adjusted *p* < 0.001.

Micro-computed tomography (micro-CT) three-dimensional reconstruction and morphometric analyses demonstrated marked trabecular bone loss in the femurs of exposed mice (Fig. 1*B*). Compared with controls, the μG, 1 Gy and μG & 1 Gy groups exhibited significantly reduced bone volume fraction (BV/TV), trabecular number (Tb.N) and trabecular thickness (Tb.Th), whereas trabecular separation (Tb.Sp) was significantly increased, indicating deterioration of trabecular microarchitecture. Consistently, bone mineral density (BMD) was markedly decreased in these groups. The GFD-treated groups showed shifts in trabecular bone parameters toward control levels (Fig. 1*C*).

Due to the cross-association between the skeletal and hematopoietic systems, we next analyzed the hematological parameters in mouse peripheral blood. Simulated space conditions were associated with marked hematological alterations. Radiation exposure induced dose-associated reductions in white blood cell lineages: total white blood cell count (WBC), particularly mid cell count (MID) and granulocyte count (GRAN), declined with increasing radiation dose (Fig. 1*D*). Detailed statistical results are provided in Supplemental Table S2.

### The Landscape of the Proton Irradiation-Associated Humoral Immunity

To explore the effects of proton irradiation on humoral immunity, we performed high-throughput autoantigen microarrays to characterize radiation-related alterations of the autoantibody reactome (Fig. 2*A*). At the nominal significance threshold of *p* < 0.05, we identified 20, 149, and 367 IgM autoantibody candidates in the Control *versus* 1 Gy, Control *versus* 5 Gy, and 1 Gy *versus* 5 Gy comparisons, respectively. Similarly, 132, 226, and 220 exploratory IgG autoantibody candidates were identified across the same comparisons (Fig. 2, *B* and *C*). After BH correction, 205 IgM and 1 IgG autoantibodies remained significant in the 1 Gy *versus* 5 Gy comparison (BH-adjusted *p* < 0.05) and were considered FDR-supported findings. Details of these differential autoantibodies are provided in Supplemental Table S3.

**Fig. 2:**
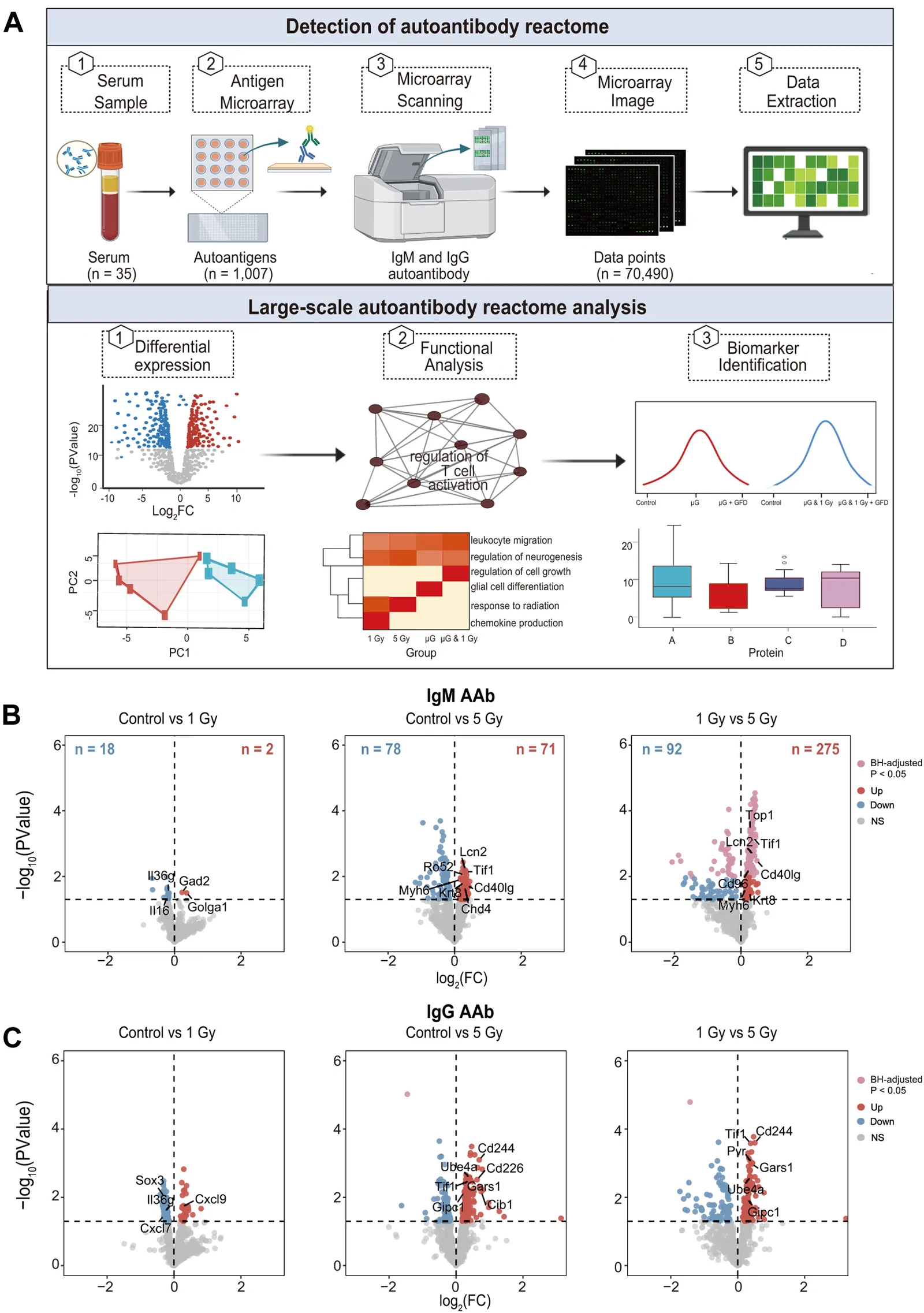
**Effect of proton irradiation on the autoantibody reactome in mice.***A*, schematic overview of the experimental workflow. *Top*: Analysis of serum samples from 35 mice on autoantigen microarrays (1007 autoantigens each) for IgM and IgG autoantibodies yielded 70,490 autoantibody reactivity measurements. *Bottom*: The workflow includes differential analysis, functional enrichment, trend analysis and identification of candidate autoantibody signatures. *B* and *C*, Volcano plots of candidate IgM (*B*) and IgG (*C*) autoantibodies across the indicated comparisons (limma, nominal *p* < 0.05). *Red* and *blue dots* indicate upregulated and downregulated autoantibodies, respectively; *gray dots* indicate non-significant changes; *pink dots* indicate FDR-supported autoantibodies. *Black labels* denote literature-matched candidates. BH-adjusted *p* values and effect sizes are provided in Supplemental Table S3.

Gene Ontology (GO) analysis revealed exploratory enrichment of radiation-associated target autoantigens in processes related to lymphocyte activation and proliferation, cytokine production, and oxidative stress-associated cell death (Fig. 3, *A* and *C* and Supplemental Table S4). Furthermore, tissue localization revealed that autoantigens corresponding to these differential autoantibodies were prominently represented among proteins associated with the brain, lymphoid tissue and skeletal muscle (Fig. 3, *B* and *D* and Supplemental Table S5).

**Fig. 3:**
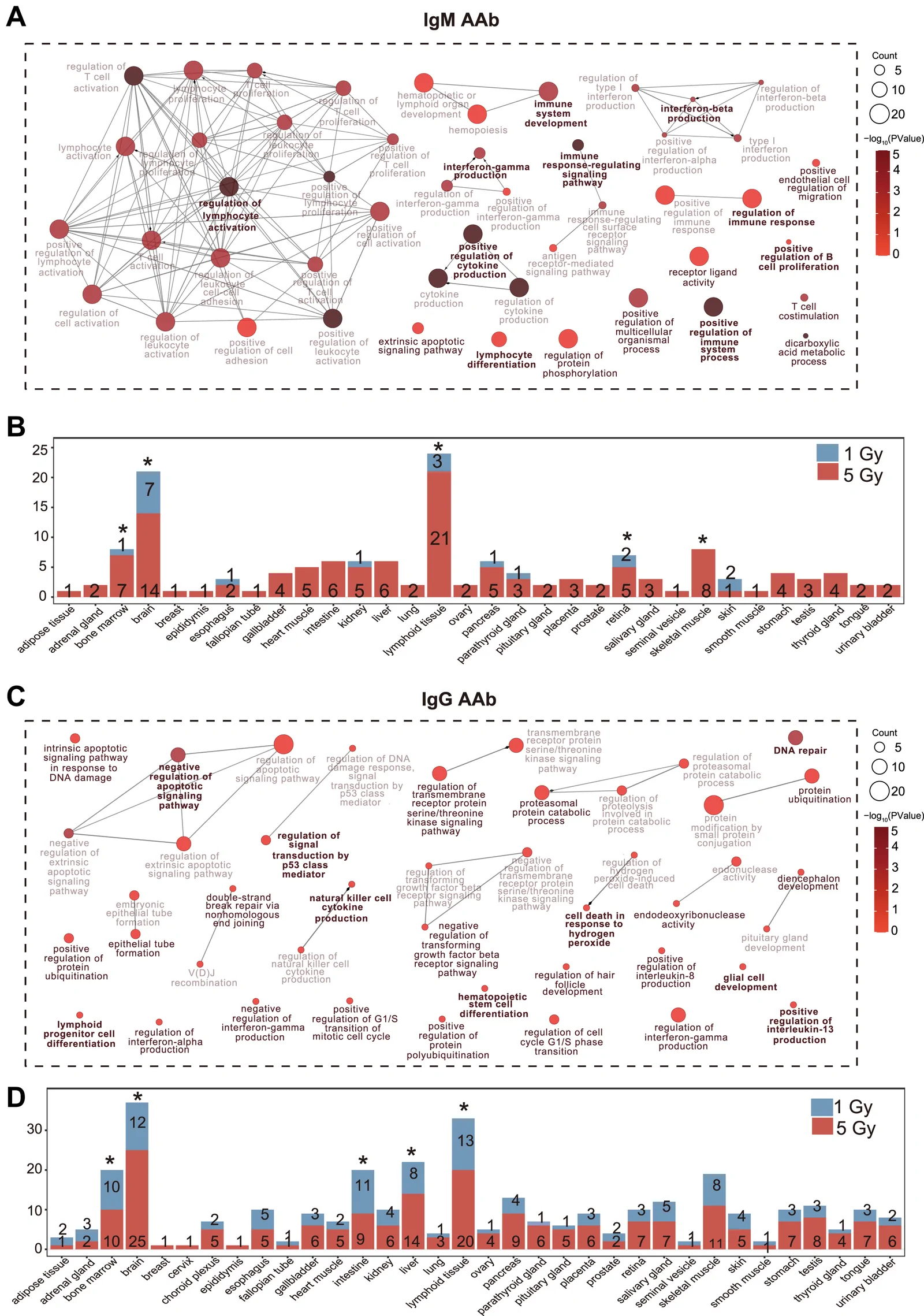
**Pathway analysis of differential autoantibodies following proton irradiation.***A* and *C*, network visualization of enriched biological processes associated with autoantigens corresponding to exploratory IgM (*A*) and IgG (*C*) autoantibody candidates autoantibodies, using the 1007 autoantigens included in the microarray as the background set. Node size represents the number of differential autoantigens enriched in each biological process. *Edge thickness* indicates the degree of autoantigen overlap between connected biological processes and *colorintensity* represents −log_10_(*p*-value). BH-adjusted *p* values and effect sizes are provided in Supplemental Table S4. *B* and *D*, tissue distribution of autoantigens corresponding to exploratory IgM (*B*) and IgG (*D*) autoantibody candidates. ∗Top five tissues with the highest abundance of radiation-associated autoantigens.

### Identification of Autoantibodies Correlated with Proton Irradiation Dose

To investigate the association between radiation dose and autoantibody levels, exploratory trend analysis identified 38 IgM and 57 IgG autoantibody candidates showing positive trends across 0, 1 and 5 Gy proton irradiation (Fig. 4, *A* and *B* and Supplemental Table S6). The corresponding target autoantigens showed FDR-supported enrichment in pathways related to cell adhesion molecule interactions and the intestinal immune network for IgA production (Fig. 4, *C* and *D* and Supplemental Table S7). The implicated molecules included co-stimulatory factors (CD86, CD40, CD80) and cytokines (IFNA4, IL25). We further selected a subset of key dose-associated candidate autoantibodies positively correlated with proton radiation dose (Fig. 4, *E* and *G*), which were clearly labeled in the volcano plots (Fig. 2, *B* and *C*). Of note, we detected IgM autoantibodies against LCN2, a protein previously reported as a marker for irradiation-induced damage (29). Box plots showing the normalized autoantibody reactivity levels of selected dose-associated candidate autoantibodies are presented in Figure 4, *F* and *H*, with the corresponding statistical details provided in Supplemental Table S8.

**Fig. 4:**
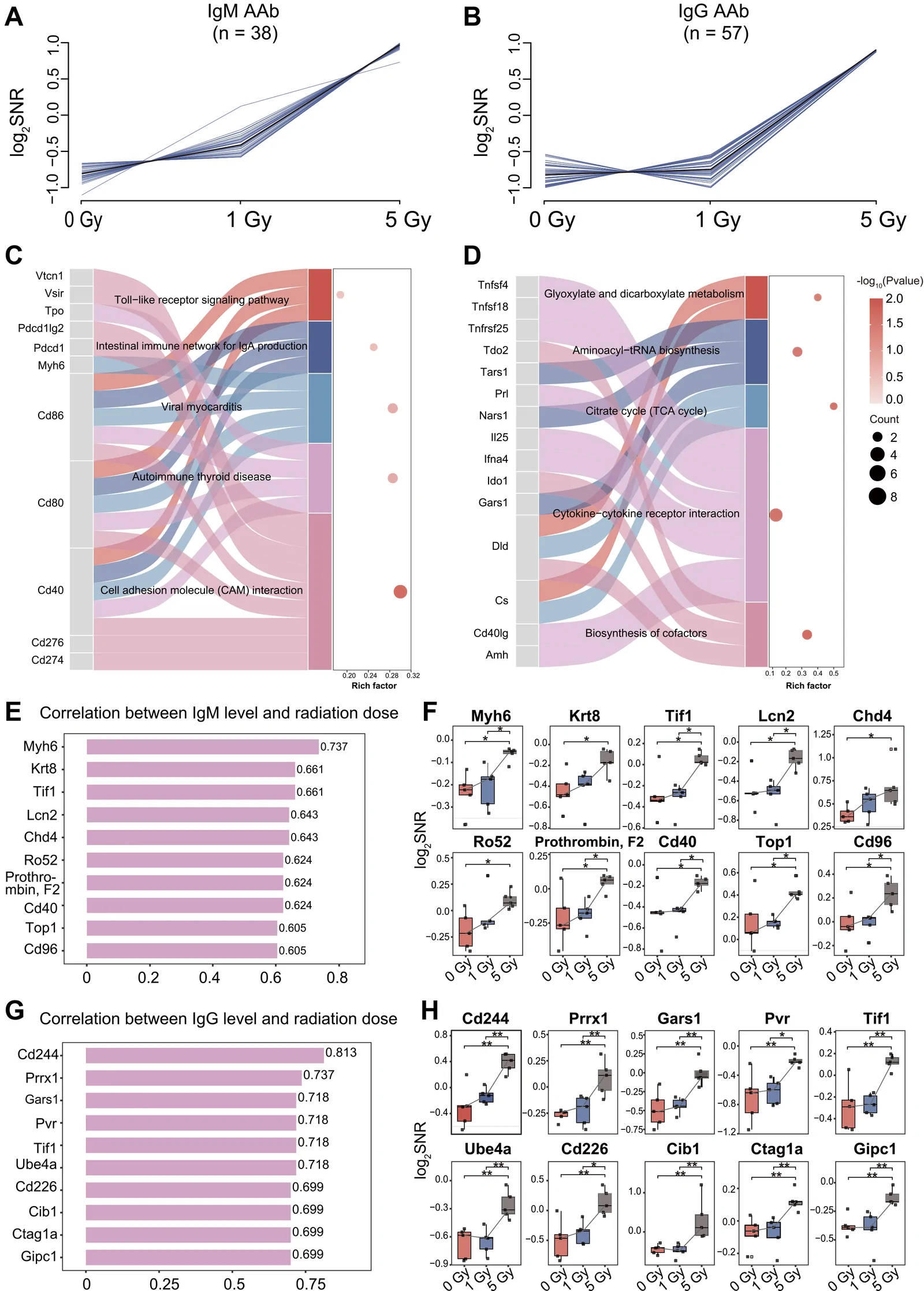
**Proton irradiation dose-associated autoantibodies.***A* and *B*, exploratory trend analysis of IgM (*A*) and IgG (*B*) autoantibodies showing dose-associated upregulation with increasing radiation doses (0 Gy, 1 Gy and 5 Gy). *Numbers* indicate the count of autoantibodies exhibiting this trend. *C* and *D*, KEGG analysis of dose-associated IgM (*C*) and IgG (*D*) autoantibodies using the 1007 microarray autoantigens as the background. *Dot size* indicates autoantibody count, *color* indicates −log_10_(*p*-value), and the x-axis shows the rich factor. BH-adjusted *p* values are provided in Supplemental Table S7. *E* and *G*, Pearson correlation analysis was performed to evaluate the association between autoantibody levels and radiation dose. *F* and *H*, *Box plots* showing the normalized autoantibody reactivity levels (log_2_-transformed SNR) of representative dose-associated candidate autoantibodies in the 0 Gy, 1 Gy and 5 Gy groups. Statistical significance was assessed by one-way ANOVA followed by emmeans pairwise contrasts with Benjamini-Hochberg adjustment. ∗BH-adjusted *p* < 0.05, ∗∗BH-adjusted *p* < 0.01, ∗∗∗BH-adjusted *p* < 0.001.

### The Landscape of the Microgravity-Associated Humoral Immunity

To assess the impact of μG on humoral immunity, we compared the μG group with controls. At the nominal significance threshold of *p* < 0.05, 87 IgM and 68 IgG autoantibodies were identified as nominally significant candidates (Fig. 5, *A* and *D*). Similarly, comparison of the μG & 1 Gy group with controls identified 104 IgM and 69 IgG nominally significant candidates (Fig. 5, *B* and *E*). Compared with the 1 Gy group, the μG & 1 Gy group exhibited upregulation of 222 IgM and 79 IgG autoantibodies at nominal *p* < 0.05, which were considered exploratory candidate signatures associated with combined exposure (Fig. 5, *C* and *F*). Details of these differential autoantibodies are provided in Supplemental Table S9.

**Fig. 5:**
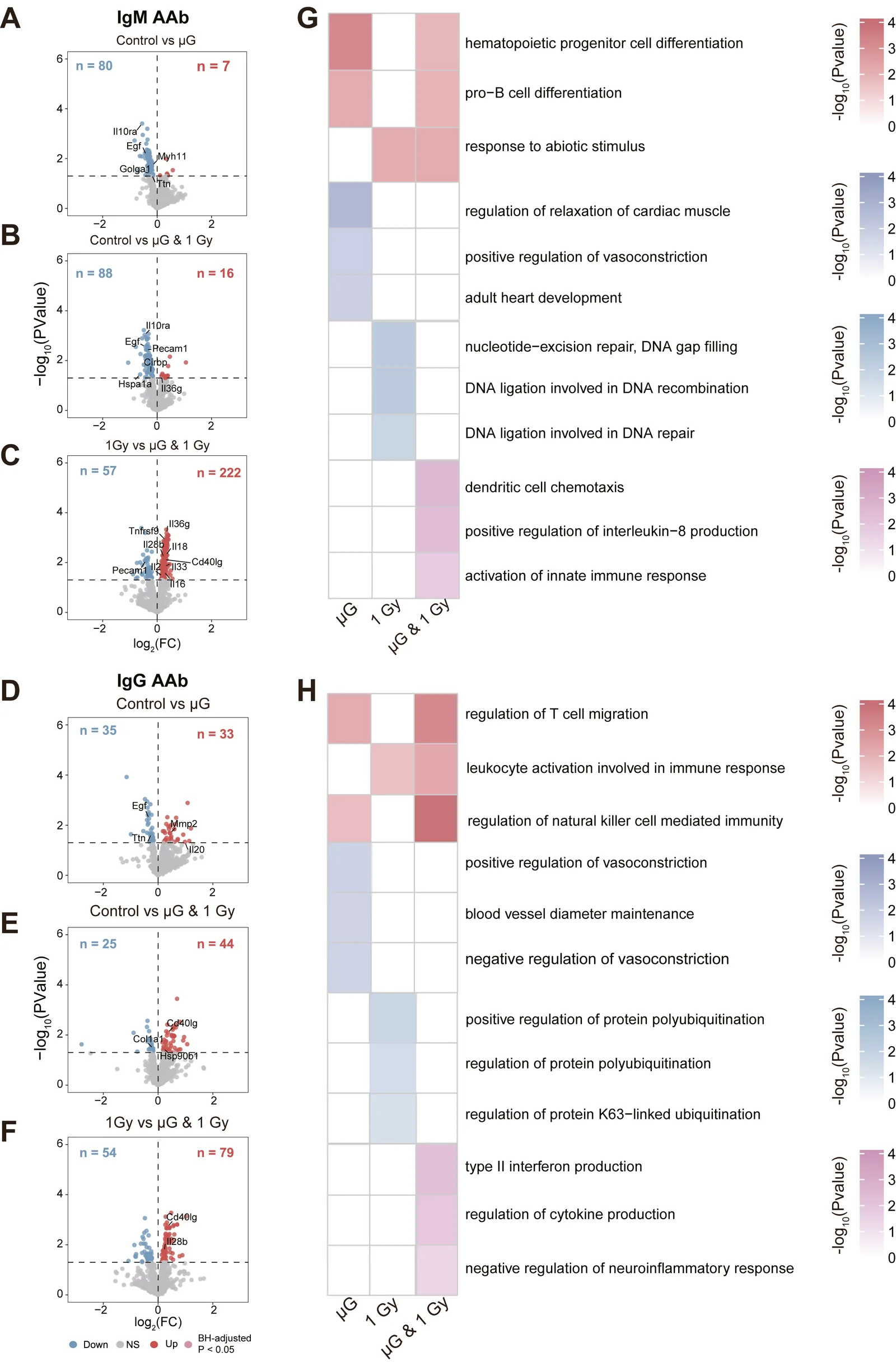
**Autoantibody profiling under simulated microgravity, proton irradiation and combined exposure.***A–C*, Volcano plots of candidate IgM autoantibodies in the Control *versus* μG (*A*), Control *versus* μG & 1 Gy (*B*) and 1 Gy *versus* μG & 1 Gy (*C*) comparisons (limma, nominal *p* < 0.05). *Red* and *blue dots* indicate upregulated and downregulated autoantibodies, respectively; *gray dots* indicate non-significant changes; *pink dots* indicate FDR-supported autoantibodies. *Black labels* denote literature-matched candidates. BH-adjusted *p* values and effect sizes are provided in Supplemental Table S9. *D–F*, Volcano plots showing candidate IgG autoantibodies for the corresponding comparisons. *G* and *H*, GO enrichment analysis of differential IgM (*G*) and IgG (*H*) autoantibodies in the μG, 1 Gy and μG & 1 Gy groups *versus* Control, using the 1007 microarray autoantigens as the background. *Color* indicates −log_10_(*p*-value); BH-adjusted *p* values are provided in Supplemental Table S11.

Previous spaceflight and ground-based simulation studies reported changes in multiple proteins, including EGF, PECAM1, HSPA1A, CD40L and COL1A1. Corresponding autoantibodies were detected in the exposure groups (Supplemental Table S10). Among the 14 proteins matched to previous studies, 50% showed previously reported protein-level alterations with directions consistent with the corresponding autoantibody changes observed in the present study.

GO analysis showed that the target autoantigens were enriched in immune cell-mediated processes, including regulation of natural killer cell-mediated immunity, leukocyte activation involved in immune response and regulation of T cell migration, consistent with previously reported spaceflight-associated alterations in immune cell function (30). However, the enrichment patterns differed markedly across conditions. In the μG group, target autoantigens were enriched in processes related to cardiovascular development and function, particularly the regulation of vasoconstriction and cardiac muscle relaxation, whereas the 1 Gy group showed enrichment in DNA damage repair and protein polyubiquitination. In the μG & 1 Gy group, target autoantigens were enriched in processes related to innate immune activation and inflammatory regulation (Fig. 5, *G* and *H*). These results were therefore interpreted as exploratory, and detailed information is provided in Supplemental Table S11.

Consistent with these enrichment patterns, tissue localization analysis suggested that μG-associated autoantibody signatures were mainly represented by brain- and cardiac tissue-associated proteins. Meanwhile, co-exposure to microgravity and proton irradiation was associated with more prominent autoantibody signatures enriched for lymphoid tissue-associated proteins (Supplemental Fig. S2*C* and Supplemental Table S12).

### Glutamine-Free Diet-Associated Modulation of Autoantibody Profiles Under Simulated Microgravity and Combined Exposure

To explore GFD-associated changes in μG-related autoantibody profiles, exploratory trend analysis showed that in the μG + GFD group, 58% (61/105) of differential IgM and 52% (43/82) of differential IgG showed shifts toward the levels observed in the control group (Supplemental Fig. S3, *A* and *B*).

Subsequent clustering analysis further divided the autoantibodies showing GFD-associated shifts toward control levels (Supplemental Table S13). Exploratory trend analysis revealed that IgM Cluster three and IgG Cluster five showed the most pronounced shifts toward control levels (Fig. 6, *A* and *C*). Kyoto Encyclopedia of Genes and Genomes (KEGG) analysis indicated that IgM Cluster three was enriched in cell motility-related pathways, including motor proteins, regulation of actin cytoskeleton and cytoskeleton in muscle cells, whereas IgG Cluster five was primarily enriched in pathways related to the endocrine and excretory systems, such as the renin–angiotensin system and vasopressin-regulated water reabsorption (Fig. 6, *B* and *D*). These results were therefore interpreted as exploratory and detailed information is provided in Supplemental Table S14. IgG Cluster five was identified as a representative autoantibody cluster showing GFD-associated shifts toward control levels under μG exposure (Fig. 6*E* and Supplemental Table S15).

**Fig. 6:**
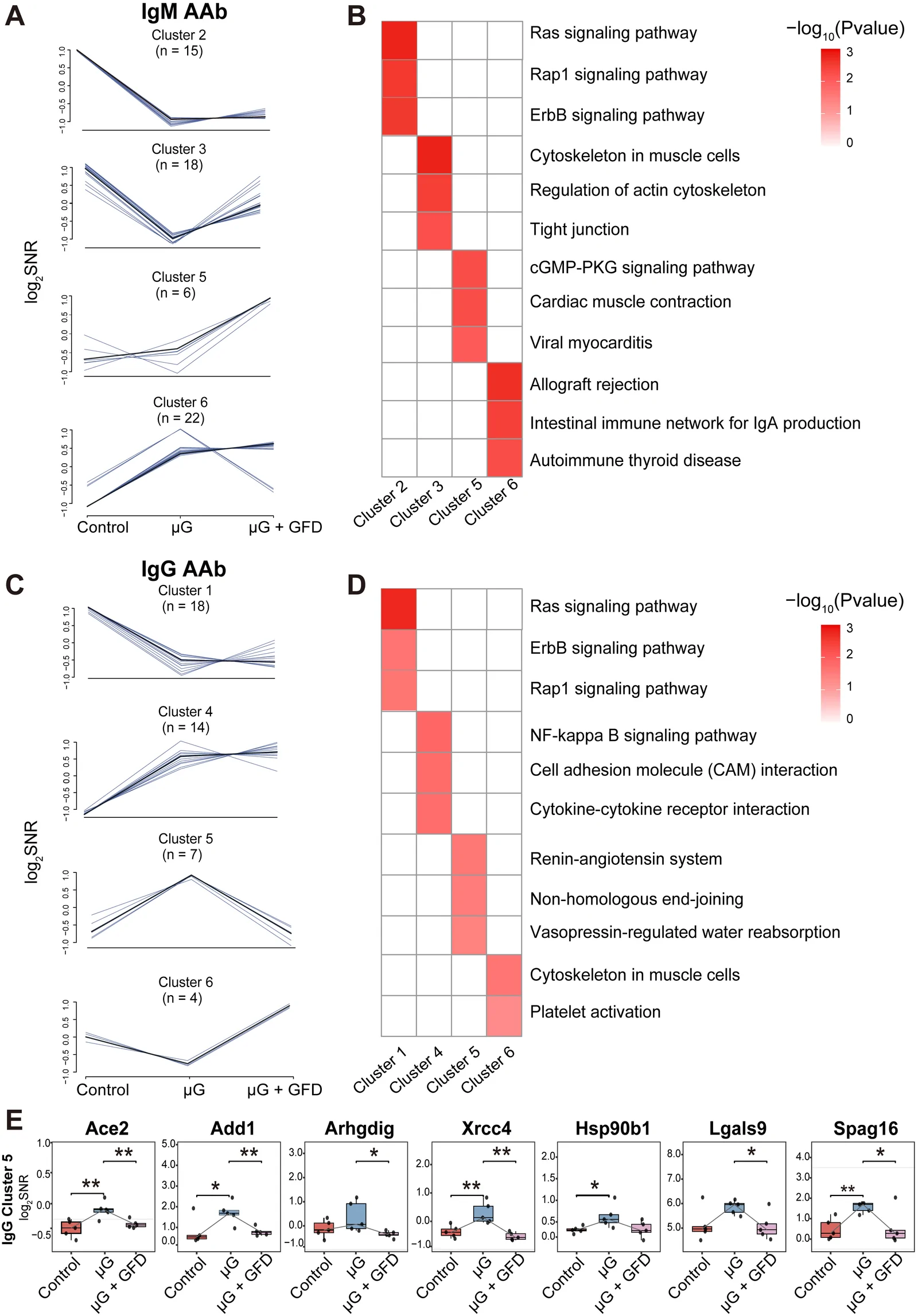
**Glutamine-free diet-associated modulation of microgravity-related autoantibody profiles.***A* and *C*, exploratory trend analysis of differential IgM (*A*) and IgG (*C*) autoantibody clusters in the Control, μG and μG + GFD groups; *numbers* represent the count of antibodies in each cluster. *B* and *D*, KEGG enrichment heatmaps of clusters of exploratory IgM (*B*) and IgG (*D*) autoantibody candidates using the 1007 microarray autoantigens as the background. *Color* indicates -log_10_(*p*-value); BH-adjusted *p* values are provided in Supplemental Table S14. *E*, *Box plots* showing the normalized autoantibody reactivity levels (log_2_-transformed SNR) of representative autoantibodies in IgG Cluster five among the Control, μG and μG + GFD groups. Statistical significance was assessed by one-way ANOVA followed by emmeans pairwise contrasts with Benjamini-Hochberg adjustment. ∗BH-adjusted *p* < 0.05, ∗∗BH-adjusted *p* < 0.01, ∗∗∗BH-adjusted *p* < 0.001.

We next assessed GFD-associated modulation under the μG & 1 Gy condition. GFD treatment was associated with shifts toward control levels in 73% (197/268) of differential IgM and 61% (52/85) of differential IgG (Supplemental Fig. S4, *A* and *B*). Similarly, cluster analysis of these differential antibodies under the μG & 1 Gy condition categorized them into six clusters (Supplemental Table S16). Among them, IgM Cluster 6 and IgG Cluster 6 showed the most pronounced shifts toward control levels (Fig. 7, *A* and *C*). KEGG analysis revealed that IgM Cluster 6 was mainly enriched in pathways related to neurodegenerative diseases and the immune system, whereas IgG Cluster 6 was primarily enriched in pathways involving signaling molecules and interactions (Fig. 7, *B* and *D*). These enrichment results were therefore interpreted as exploratory. Detailed information is provided in Supplemental Table S17. Moreover, IgG Cluster 6 was identified as a representative exploratory candidate associated with GFD intervention under μG & 1 Gy exposure (Fig. 7E and Supplemental Table S18).

**Fig. 7:**
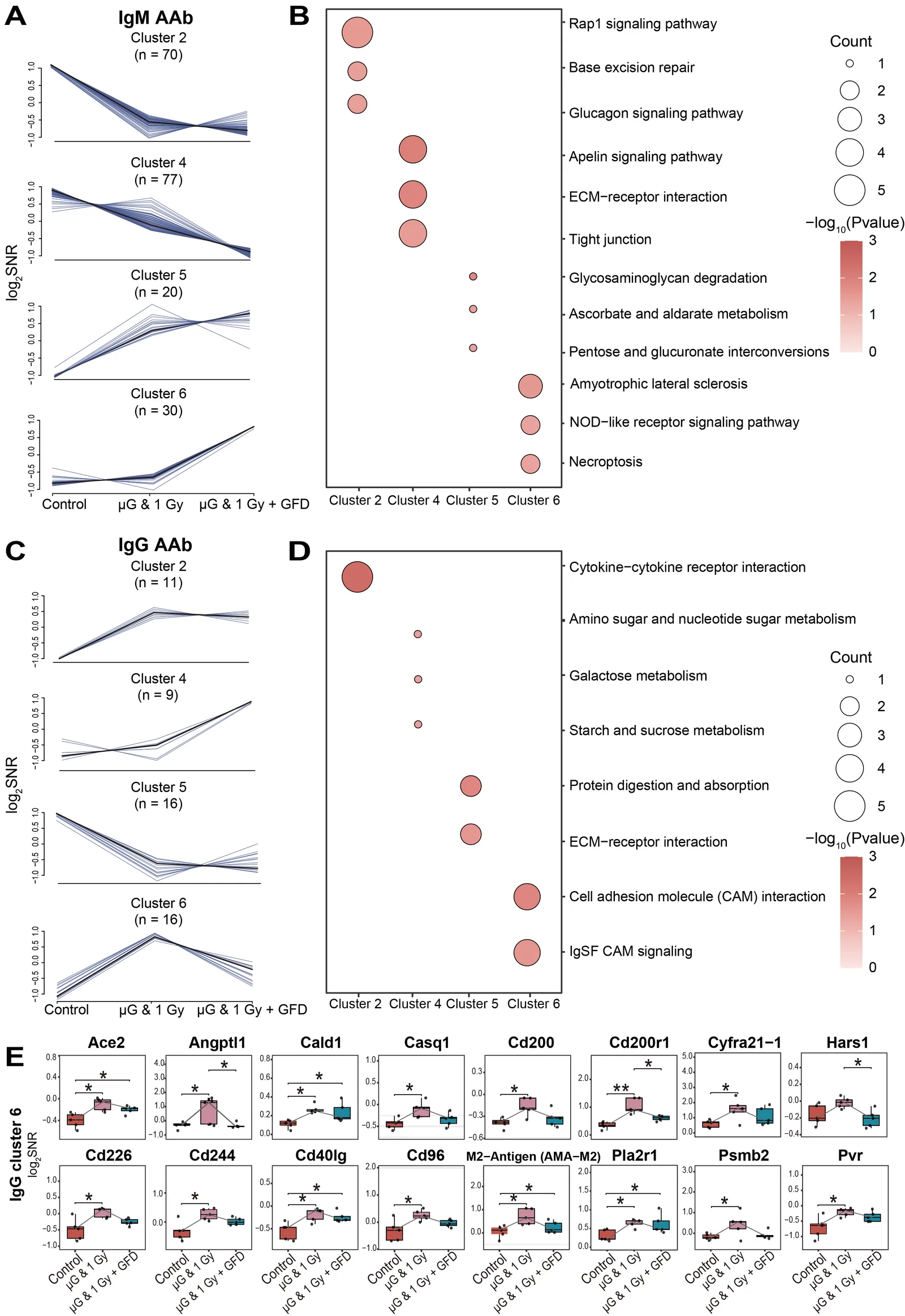
**Glutamine-free diet-associated modulation of autoantibody profiles under combined microgravity and 1 Gy proton irradiation.***A* and *C*, exploratory trend analysis of differential IgM (*A*) and IgG (*C*) autoantibody clusters in the Control, μG & 1 Gy and μG & 1 Gy + GFD groups; numbers represent the count of autoantibodies in each cluster. *B* and *D*, KEGG enrichment heatmaps of clusters of exploratory IgM (*B*) and IgG (*D*) autoantibody candidates using the 1007 microarray autoantigens as the background. *Color* indicates −log_10_(*p*-value); BH-adjusted *p* values are provided in Supplemental Table S17. *E*, *Box plots* showing the normalized autoantibody reactivity levels (log_2_-transformed SNR) of representative autoantibodies in IgG Cluster 6 among the Control, μG & 1 Gy and μG & 1 Gy + GFD groups. Statistical significance was assessed by one-way ANOVA followed by emmeans pairwise contrasts with Benjamini-Hochberg adjustment. ∗BH-adjusted *p* < 0.05, ∗∗BH-adjusted *p* < 0.01, ∗∗∗BH-adjusted *p* < 0.001.

### Assay-Level Replication of Selected Candidate Autoantibody Reactivity

To assess the robustness of selected candidate signals across array fabrication and experimental batches, 217 prioritized autoantigens were remeasured using an independently fabricated customized autoantigen microarray. This analysis reproduced three radiation dose-responsive candidate signals, 11 μG-associated candidate signals, seven candidate signals associated with combined μG + 1 Gy exposure, and one GFD-associated candidate signal (Supplemental Fig. S5). In addition, seven GFD-associated autoantibodies showed concordant directional changes between the discovery and customized microarrays but did not reach the BH-adjusted significance threshold (Supplemental Fig. S6).

Because the same animals and serum samples were used in both experiments, these findings represent assay-level replication rather than independent biological validation. Candidates that did not meet the BH-adjusted significance threshold were therefore considered exploratory signals.

## Discussion

Although spaceflight-induced immune dysfunction is largely attributed to μG and radiation, how these stressors affect humoral immunity remains poorly defined (31). In this study, we systematically profiled humoral immune responses under simulated spaceflight conditions and identified distinct and overlapping alterations associated with μG and proton irradiation.

Under simulated spaceflight conditions, co-exposure to μG and proton irradiation was associated with pronounced alterations in humoral immune responses, consistent with previous studies reporting enhanced adverse effects under combined radiation and simulated microgravity conditions (32, 33). Previous studies have shown that μG can disrupt cellular structure, alter apoptosis-related processes and impair immune organ function, whereas radiation can induce DNA damage, oxidative stress and cell death. These stressor-associated biological processes may converge on inflammatory regulation, oxidative stress responses, DNA repair and apoptosis-related pathways, potentially contributing to altered immune homeostasis (34).

We identified candidate autoantibody signatures associated with μG and radiation, among which 38 IgM and 57 IgG candidate autoantibody signatures showed dose-related trends across 0, 1, and 5 Gy proton irradiation, including anti-LCN2 IgM and anti-Ro52 IgM. LCN2 is known to play a pivotal role in iron homeostasis, with intracellular iron levels closely linked to reactive oxygen species (ROS) production (35, 36). Ionizing radiation induces DNA double-strand breaks and promotes ROS generation (37, 38). As a neutrophil-associated acute-phase protein, LCN2 has been implicated in tissue injury through its involvement in iron metabolism, oxidative stress and ferroptosis (39, 40). Similarly, it has been reported that the expression of Ro52 in skin keratinocytes is upregulated by UV radiation and may undergo conformational changes or cleavage, exposing cryptic epitopes that break immune tolerance and promote the production of anti-Ro52 autoantibodies in genetically susceptible individuals (41, 42). Notably, proton irradiation-associated target antigens showed enrichment among proteins associated with multiple tissue compartments, including lymphoid tissues, brain and bone marrow. Such widespread distribution may reflect the systemic nature of radiation-associated immune perturbations. Previous studies have shown that high-dose radiation (>1 Gy) can cause acute necrotic damage to lymphoid tissues, leading to massive lymphocyte depletion and immune collapse, which could underlie the broad distribution of these autoantibodies (43, 44). Nevertheless, tissue-enrichment analysis based on autoantibody targets does not directly demonstrate tissue injury or functional impairment. These findings should therefore be interpreted as altered humoral recognition patterns rather than direct evidence of radiation-induced tissue damage. Future studies integrating tissue transcriptomics, proteomics, histological analysis, imaging and functional assays are needed to determine the biological consequences of these autoantibody alterations. Among the proton irradiation-associated exploratory candidates, several representative autoantibody signatures, including anti-TIF1 IgM, anti-GIPC1 IgG and anti-TIF1 IgG, showed concordant changes in the assay-level microarray-batch replication experiment using an independently fabricated array batch, supporting their reproducibility across microarray batches.

Interestingly, comparison with previously reported plasma protein changes from spaceflight and ground-based simulation studies revealed partial concordance, with 7 of 14 matched proteins (50%) showing changes consistent with the corresponding autoantibody alterations under μG and μG & 1 Gy. Differences in species, experimental platforms, and detection strategies may partly account for the incomplete concordance. Moreover, 11 μG-associated signatures and 7 μG & 1 Gy-associated signatures identified in the discovery phase were reproduced in the microarray-batch replication experiment, supporting assay-level reproducibility rather than independent biological validation. Further validation in larger independent cohorts using orthogonal assays is therefore required. These findings suggest that certain autoantibodies may serve as candidate signatures of space exposure. However, it remains unclear whether exposure-associated protein changes drive corresponding autoantibody alterations or whether autoantibody changes themselves contribute to pathological effects. Future studies should further investigate their functional relevance and potential role in maintaining immune homeostasis under spaceflight conditions.

μG and proton irradiation were associated with distinct yet interconnected patterns of humoral immune alteration. Autoantibody targets altered under μG conditions were enriched for cardiovascular-associated proteins, consistent with previous reports of physiological ventricular atrophy, increased arterial stiffness and altered blood-flow distribution under μG conditions (45, 46). High flow and shear stress can induce endothelial and smooth muscle cells to express matrix metalloproteinase-2 (MMP-2), which contributes to increased vascular stiffness (47, 48). However, altered anti-MMP-2 autoantibody reactivity under μG exposure may reflect a humoral immune response associated with cardiovascular-related adaptations. Whether these autoantibodies contribute to, compensate for or simply reflect μG-associated physiological changes remains unclear and requires further investigation. In contrast, autoantibody targets altered under 1 Gy proton irradiation were enriched for proteins associated with DNA repair and protein ubiquitination. Ionizing space radiation is known to cause oxidative DNA damage and activate oxidative stress responses (49, 50). This oxidative stress can disrupt immune homeostasis. Under combined exposure to μG and 1 Gy proton irradiation, autoantibody targets showed prominent enrichment in processes related to innate immune activation and inflammatory regulation. One possible interpretation is that radiation-associated protein damage, potentially accompanied by altered ubiquitin-proteasome function, may converge with μG-related stress at the level of inflammatory regulation. Damaged proteins or protein aggregates could act as endogenous danger signals and may contribute to cytokine-related processes involving IL-16, IL-18 and IL-33. This interpretation is consistent with previous reports of inflammatory alterations under spaceflight and simulated microgravity conditions (51, 52).

Importantly, GFD-treated groups showed shifts in selected bone, hematological and autoantibody parameters toward control levels under the tested conditions, suggesting that glutamine metabolism may contribute to the immune and skeletal alterations induced by simulated space conditions. This finding is consistent with our previous work, which demonstrated that mechanical unloading enhances glutamine uptake and catabolism in monocytes and that disrupting this process can induce apoptosis under pathological conditions via the XIAP/Diablo axis (23). One possible explanation is that glutamine availability may influence the survival and function of pathologically activated cells under simulated space conditions, although this mechanism was not directly examined in the present study. Additionally, IgG Cluster five in the μG group and IgG Cluster 6 in the μG & 1 Gy group were identified as exploratory candidate autoantibody clusters associated with GFD-related changes.

Several limitations should be acknowledged. First, the relatively small group size (n = 5 per group) limited statistical power, and nominally significant signals from the 1007-autoantigen screen were therefore considered exploratory unless supported by BH-adjusted *p* < 0.05. Although selected candidates were reproduced using an independently fabricated customized microarray, the use of the same biological samples indicates assay-level reproducibility rather than independent biological validation. Larger independent cohorts and orthogonal assays will be required to further establish the biological relevance and generalizability of these autoantibody signals. Second, the acute single-dose proton irradiation model does not fully reproduce the chronic, low-dose rate, mixed-field radiation environment encountered during spaceflight. Third, body weight and food intake were not systematically monitored, and the lack of appropriate dietary controls may influence the interpretation of GFD-associated findings.

Our findings provide an exploratory framework for investigating humoral immune alterations under simulated space conditions and support further evaluation of dietary modulation as a potential countermeasure.

## Conclusion

We characterized the autoantibody reactome under simulated space conditions and found that combined μG and proton irradiation exposure was associated with a distinct pattern of humoral immune alterations, including both exposure-specific and overlapping autoantibody signatures. Moreover, GFD was associated with shifts in selected bone, hematological and autoantibody parameters toward control levels under the tested conditions. These findings provide a framework for investigating humoral immune alterations under simulated space-related stress and support further investigation of dietary interventions as potential countermeasures. Future studies incorporating larger cohorts, appropriate dietary control groups, and improved simulation models are needed to validate these findings and clarify the underlying mechanisms.

## Data Availability

The autoantibody microarray data have been deposited to the ProteomeXchange Consortium (http://proteomecentral.proteomexchange.org) via the iProX partner repository with the dataset identifier PXD077126 (53, 54).

## Supplemental Data

This article contains supplemental data.

## Conflict of Interest

The authors declare that they have no conflicts of interest with the contents of this article.
