# Supplementary material for: Autoantibody Reactome Profiling Reveals Distinct Humoral Immune Signatures Induced by Simulated Microgravity, Radiation, and Combined Exposure in Mice
Source: Mol Cell Proteomics. 2026 Sep 18;25(11):101665. doi: 10.1016/j.mcpro.2026.101665 (PMC13691153; doi:10.1016/j.mcpro.2026.101665)
Supplement: Supplementary file 1 [file mmc19.docx]

Supplementary material

Autoantibody reactome profiling reveals distinct humoral immune signatures induced by simulated microgravity, radiation and combined exposure in mice

Lining Wu^1,3#^, Kaipeng Zheng²^#^, Bomiao Yu^3#^, Bingxin Gao^2#^, Pancheng Xiao^1,3#^, Saiya Wang⁴, Yanjun Li³, Ruimin Liu³, Xiaomei Zhang^3^, Mansheng Li³*, Chun-Ping Cui³*, Weiming Tian²*, Xiaobo Yu^1,3^*

**Affiliations**

^1^School of Basic Medical Sciences, Anhui Medical University, Hefei 230022, Anhui, China

^2^School of Life Science and Technology, Harbin Institute of Technology, Harbin 150001, Heilongjiang, China

^3^State Key Laboratory of Medical Proteomics, Beijing Proteome Research Center, National Center for Protein Sciences (Beijing), Beijing Institute of Lifeomics, Beijing 102206, China

^4^ProteomicsEra Medical Co., Ltd., Beijing 102206, China

\# These authors contributed equally to this work.

*Corresponding Authors

Email: limansheng133@163.com (M.L.); cui_chunping2000@aliyun.com (C.C.); tianweiming@hit.edu.cn (W.T.); xiaoboyu@vip.126.com (X.Y.)

The supplementary contains:

Supplementary Text

Figures S1 to S6

References (1 to 5)

Other Supplementary Tables for this manuscript include:

| Table S1 | Mice model information |
| --- | --- |
| Table S2 | Statistical analysis of bone microarchitecture and hematological parameters |
| Table S3 | Differential antibodies in pairwise comparisons among 1 Gy, 5 Gy and control groups |
| Table S4 | Biological Process enrichment analysis of radiation-responsive autoantibodies |
| Table S5 | Tissue localization analysis of differential autoantibodies under 1 Gy and 5 Gy exposure |
| Table S6 | Radiation dose-associated autoantibody levels |
| Table S7 | KEGG analysis of differential autoantibodies in the 0 Gy, 1 Gy and 5 Gy groups |
| Table S8 | Statistical details of radiation dose-correlated autoantibodies |
| Table S9 | Differential autoantibodies in the Control vs μG, Control vs μG & 1 Gy and 1 Gy vs μG & 1 Gy comparisons |
| Table S10 | Humoral immune responses of autoantibodies corresponding to spaceflight-associated proteins |
| Table S11 | Biological Process analysis of differential autoantibodies in the μG, 1 Gy and μG & 1 Gy groups |
| Table S12 | Tissue localization analysis of differential autoantibodies in μG and μG & 1 Gy exposure |
| Table S13 | Clustering analysis of differential autoantibodies in control, μG and μG + GFD groups |
| Table S14 | KEGG analysis of differential autoantibodies in the Control, μG and μG + GFD groups |
| Table S15 | Statistical analysis of representative differential autoantibodies affected by GFD intervention under μG exposure |
| Table S16 | Clustering analysis of differential autoantibodies in control, μG & 1 Gy and μG & 1 Gy + GFD groups |
| Table S17 | KEGG analysis of differential autoantibodies in the Control, μG & 1 Gy and μG & 1 Gy + GFD groups |
| Table S18 | Statistical analysis of representative differential autoantibodies affected by GFD intervention under combined μG and 1 Gy exposure |

**Supplementary Methods**

**Mouse models:** All animal experimental procedures were performed in accordance with the guidelines of the Biosafety and Ethics Committee of Harbin Institute of Technology (Ethics Approval No. IACUC-2026015). Eight-week-old male specific pathogen-free (SPF) C57BL/6J mice (20 ± 2 g) were purchased from Liaoning Changsheng Biotechnology Co., Ltd. (Liaoning, China). Mice were housed under controlled SPF conditions at 22 ± 2°C and 55% ± 10% relative humidity with a 12 h light/12 h dark cycle and free access to food and water. After 7 days of acclimatization, mice were randomly assigned to seven groups (n = 5 per group): Control, μG, 1 Gy, 5 Gy, μG & 1 Gy, μG + GFD and μG & 1 Gy + GFD. Control mice were maintained under the same environmental and housing conditions throughout the experimental period but were not subjected to hindlimb unloading or proton irradiation. Body weight and food intake were not systematically monitored during the experimental period.

**Establishment of the microgravity model:** Simulated microgravity was induced by tail suspension in mice (1,2). After disinfection with 75% ethanol, mouse tails were wrapped and secured along their longitudinal axis using medical breathable adhesive tape. The distal end of the tape was attached via a hook to a suspension apparatus fixed to the cage top. The suspension height was adjusted such that the hind limbs were completely elevated off the cage floor, with the longitudinal body axis forming a 30° angle with the horizontal plane, while the forelimbs remained freely movable and weight-bearing on the cage floor. Continuous suspension was maintained for 28 days. Tail skin integrity was monitored daily to prevent pressure necrosis and tape detachment. Specifically, mice in the μG group were fed a standard maintenance diet, whereas those in the μG + GFD group received a glutamine-free diet (Biopike, M11708).

**Establishment of mouse proton irradiation model:** Mice assigned to the 1 Gy and 5 Gy groups received single whole-body proton irradiation at doses of 1 Gy and 5 Gy, respectively (3-5). Irradiation was performed using a proton beam accelerator at the Ground-based Space Environment Simulation Facility of Harbin Institute of Technology. Mice received a single whole-body exposure to a 150 MeV proton beam generated by a cyclotron, with a total dose of 1 Gy or 5 Gy delivered at a dose rate of 0.2 Gy/min. During irradiation, mice were individually housed in ventilated plastic irradiation chambers and positioned at the center of the radiation field. After irradiation, mice in the 1 Gy and 5 Gy groups were maintained under standard housing conditions for 28 days and sampled at the same experimental endpoint as the other groups.

**Establishment of the combined proton radiation and microgravity group:** Mice assigned to the μG & 1 Gy and μG & 1 Gy + GFD groups first received 1 Gy whole-body proton irradiation. After a 24-h recovery period under standard housing conditions, mice were subjected to simulated microgravity for 28 days. Mice in the μG & 1 Gy group received a standard maintenance diet, whereas those in the μG & 1 Gy + GFD group received a glutamine-free diet.

**Whole blood collection and serum preparation:** At the end of the respective treatment periods, samples were collected from all groups. For mice in the hindlimb unloading groups, samples were collected immediately after release from suspension to minimize stress-induced interference with immune parameters. Prior to blood collection, the periorbital skin was disinfected with 75% ethanol. The scruff of the mouse’s neck was gently restrained to induce slight exophthalmos, and a non-heparinized glass capillary tube was gently inserted along the medial orbital wall from the inner canthus at an approximately 45° angle to a depth of 2-3 mm. Blood was collected into sterile 1.5 mL microcentrifuge tubes and allowed to clot at room temperature for 2 h, followed by centrifugation at 3,000 rpm for 15 min at 4 °C. The serum was collected, immediately snap-frozen in liquid nitrogen, and stored at -80 °C until further analysis. Following blood collection, mice were euthanized by cervical dislocation.

**Femur sampling:** Following euthanasia, femurs were rapidly isolated from each mouse. For mice in the hindlimb unloading groups, femur collection was performed immediately after blood collection and euthanasia to minimize reversal of unloading-induced bone phenotypic changes. Under sterile conditions, adherent muscles, tendons, and surrounding connective tissues were carefully removed from the bone surface. The isolated femurs were briefly rinsed with pre-cooled sterile phosphate-buffered saline (PBS) and blotted dry with sterile filter paper. For micro-computed tomography (micro-CT) and histomorphometric analyses, femoral specimens were fixed in 4% paraformaldehyde and stored at 4 °C.

**References**

1.	Li, H., T. Cao, W. Ding, L. Liang, G.-C. Fan, L. Qu, T. Peng (2022) Pharmacological inhibition of Rac1 attenuates myocardial abnormalities in tail-suspended mice. J Cardiovasc. Transl. Res., 15, 805–815 doi:10.1007/s12265-021-10197-7

2.	Zhao, Z., X. Wang, Y. Ma, X. Duan (2024) Atp6v1h deficiency blocks bone loss in simulated microgravity mice through the fos-jun-src-integrin pathway. Int. J. Mol. Sci., 25, 637 doi:10.3390/ijms25010637

3.	Werneth, C. M., Z. S. Patel, M. S. Thompson, S. R. Blattnig, J. L. Huff (2024) Considering clonal hematopoiesis of indeterminate potential in space radiation risk analysis for hematologic cancers and cardiovascular disease. Commun. Med., 4, 105 doi:10.1038/s43856-023-00408-4

4.	Sihver, L., S. M. J. Mortazavi (2021) Biological protection in deep space missions. J. Biomed. Phys. Eng., 11, 663–674 doi:10.31661/jbpe.v0i0.1193

5.	Pannkuk, E. L., E. C. Laiakis, T. D. Mak, G. Astarita, S. Authier, K. Wong, A. J. Fornace (2016) A lipidomic and metabolomic serum signature from nonhuman primates exposed to ionizing radiation. Metabolomics Off. J. Metabolomic Soc., 12, 80 doi:10.1007/s11306-016-1010-0

Supplementary Figures

Figure S1. Quality control and data distribution overview of the antigen microarray

(A) Tissue distribution of the autoantigens represented on the microarray based on HPA annotations. The Y-axis represents the number of autoantigens associated with each tissue, and the X-axis represents the annotated tissue types.
(B) Reproducibility of the autoantigen microarray for serological autoantibody detection. The diagonal shows the distribution of sample values, the lower-left panels show bivariate scatter plots with fitted lines, and the upper-right panels show Pearson correlation coefficients and corresponding significance levels (****P* < 0.001).
(C) Distribution of autoantibody levels across different groups (Control, μG, 1 Gy, μG & 1 Gy, 5 Gy, μG + GFD, μG & 1 Gy + GFD). The violin plot simultaneously presents the probability density (width) and median (white horizontal line) of the data, reflecting the central tendency and dispersion of autoantibody levels in different groups.

(D) PCA score plots showing the global IgM and IgG autoantibody profiling differences between the control group and each simulated space-environment exposure group, including μG, 1 Gy, μG & 1 Gy and 5 Gy. Each point represents an individual mouse, and shaded areas indicate group distributions.

(E) PCA score plots showing the effects of GFD intervention on IgM and IgG autoantibody profiles under μG or combined μG & 1 Gy exposure. Comparisons include μG versus μG + GFD and μG & 1 Gy versus μG & 1 Gy + GFD. Each point represents an individual mouse, and shaded areas indicate group distributions.

Figure S2. Tissue localization and PCA of autoantibodies under microgravity and radiation

(A) Exploratory PCA of differential IgM and IgG autoantibodies in pairwise comparisons among the Control, 1 Gy and 5 Gy groups.

(B) Exploratory PCA of differential IgM and IgG autoantibodies in pairwise comparisons among the Control, μG and μG & 1 Gy groups.

(C) Tissue localization of differential IgM and IgG autoantibodies in the μG and μG & 1 Gy groups.

Figure S3. Glutamine-free diet-associated shifts in autoantibody profiles under simulated microgravity

(A) Exploratory trend analysis of differential IgM autoantibody clusters in the Control, μG and μG + GFD groups. Each line represents the expression profile of a single autoantibody and the color gradient distinguishes the expression trend differences among distinct autoantibodies.
(B) Exploratory trend analysis of differential IgG autoantibody clusters in the Control, μG and μG + GFD groups.

Figure S4. Glutamine-free diet-associated shifts in autoantibody profiles under combined microgravity and 1 Gy proton irradiation

(A) Exploratory trend analysis of differential IgM autoantibody clusters in the Control, μG & 1 Gy and μG & 1 Gy + GFD groups. Each line represents the expression profile of a single autoantibody and the color gradient distinguishes expression trend differences among distinct autoantibodies.
(B) Exploratory trend analysis of differential IgG autoantibody clusters in the Control, μG & 1 Gy and μG & 1 Gy + GFD groups.

Figure S5. Assay-level microarray-batch replication of selected candidate autoantibody signatures

(A) Box plots showing the log₂-transformed SNR values of selected candidate autoantibody signatures reproduced using a separately fabricated microarray batch with the same serum samples. Dots represent individual samples, and lines connect group medians. Statistical significance was assessed using one-way ANOVA followed by pairwise contrasts using the emmeans package. For each autoantibody, P values from all pairwise contrasts were adjusted together using the Benjamini-Hochberg method. *BH-adjusted *P* < 0.05, **BH-adjusted *P* < 0.01, ***BH-adjusted *P* < 0.001. A candidate signature was considered reproduced when it showed a concordant direction of change with the discovery-phase result and reached BH-adjusted P < 0.05 in the corresponding comparison in the microarray-batch replication experiment.

Figure S6. Concordant trends of GFD-associated candidate autoantibody signals in the microarray-batch replication experiment

(A) Box plots showing the log₂-transformed SNR values of GFD-associated candidate autoantibody signals using a separately fabricated microarray batch with the same serum samples. Dots represent individual samples. Statistical significance was assessed using one-way ANOVA followed by pairwise contrasts using the emmeans package. For each autoantibody, the P values from all pairwise contrasts were adjusted together using the Benjamini-Hochberg method. These candidates showed concordant directional changes with the discovery-phase results but did not reach statistical significance after BH adjustment and were therefore considered trend-level evidence.
